# Above and beyond senescence and CAR T cell: advances and future perspectives

**DOI:** 10.3389/fimmu.2025.1701655

**Published:** 2025-12-16

**Authors:** Rebeca Rosas-Campos, Scarlet Arceo-Orozco, Ana Sandoval-Rodriguez, Jose Alejandro Madrigal, Juan Armendariz-Borunda

**Affiliations:** 1 Tecnologico de Monterrey, EMCS, Guadalajara, Mexico; 2 Institute for Molecular Biology in Medicine and Gene Therapy, Health Sciences University Center, University of Guadalajara, Guadalajara, Mexico; 3 Cancer Institute, University College London, London, United Kingdom

**Keywords:** aging-related diseases, CAR T cell, immunotherapy, senescence, targeting senescent cells

## Abstract

Cellular senescence is a complex biological process that contributes to the progression of age-related diseases. Senescent cells accumulate over time and secrete proinflammatory factors that disrupt tissue homeostasis and promote chronic diseases. Initially created for cancer immunotherapy, chimeric antigen receptor (CAR) T cell therapy has recently been repurposed at the preclinical level to eliminate senescent cells by recognizing senescence-associated surface markers. Recent preclinical studies have demonstrated the feasibility of anti-uPAR and anti-NKG2D CAR T cell-based approaches for eliminating senescent cells in preclinical models of fibrosis, metabolic disorders, and natural aging, resulting in reduced fibrotic burden, improved metabolic parameters, and enhanced tissue function. However, several challenges remain in their clinical applicability, including the identification of specific and universal senescence markers, potential off-target effects, and long-term safety concerns. Additionally, immunosenescence, an age-related decline in immune function, poses challenges for CAR T cell therapy in elderly patients. This review discusses recent advancements in CAR T cell therapy for senescence clearance, highlighting key molecular targets, preclinical findings, and future research directions. Despite their promise, a significant translational gap persists, and further research is required to improve the specificity, efficacy, and readiness for future human clinical trials.

## 1 Introduction

Cellular senescence is a biological process characterized by irreversible cell cycle arrest, resistance to apoptosis, and the secretion of pro-inflammatory and tissue damaging factors collectively known as the senescence-associated secretory phenotype (SASP). The progressive accumulation of senescent cells over time leads to tissue dysfunction and drives the development of multiple age-related pathologies, including sarcopenia, atherosclerosis, heart failure, chronic kidney disease, osteoporosis, macular degeneration, pulmonary insufficiency, and neurodegeneration (1).

Given the increasing interest in extending life expectancy and the central role of senescence in aging and disease, considerable research has focused on therapeutic strategies targeting senescent cells. Senotherapeutic agents include senolytics, which selectively remove senescent cells, and senomorphics, which suppress or modulate the SASP without inducing cell death (2). The combination of Dasatinib and Quercetin is among the most well characterized senolytic treatments and has demonstrated efficacy in preclinical models of age-related pathologies. Desatinib is a tyrosine kinase inhibitor, whereas Quercetin interacs with a PI3K isoform and Bcl-2 family members, and together, they induce apoptosis in senescent cells more efficiently (3). Concurrently, this combination has been tested in clinical trials for conditions such as Alzheimer’s disease, idiopathic pulmonary fibrosis, chronic kidney disease, skeletal decline, hematopoietic stem cell transplant complications, and fibrotic non-alcoholic fatty liver disease (4). Additional senolytic classes include BCL-2 family inhibitors, HSP90 inhibitors, galactose-modified prodrugs, and cardiac glycosides. Senomorphic agents include inhibitors of NF-κB, p38 MAPK, and the JAK/STAT pathway, as well as compounds such as rapamycin and metformin, which modulate the mTOR, Nrf2, and NF-κB pathways (3).

However, pharmacologic senotherapeutics may induce unintended off-target effects due to the role of many of their molecular targets in normal physiological process and immune functions. This limitation highlights the need for more precise strategies capable of remove senescent cells while minimizing adverse effects. In this context, immunotherapies, such as Chimeric antigen receptor (CAR) T cells, are particularly noteworthy for their high specificity, adaptability, and ability to be reprogrammed to target markers selectively expressed by senescent cells, rendering them a promising next generation senolytic strategy.

CAR T cell therapy harnesses advancements in gene editing and cell therapy to direct T cells against specific cell surface antigens of interest. Currently, the Food and Drug Administration Agency (FDA) have approved six CAR T cell therapies for hematological malignancies treatment (5). Eshhar and Gross were pioneers in CAR development; they fused a single-chain variable fragment (scFv), a specific antigen, to the CD3ζ chain, the common signaling component of the T-cell receptor (TCR) (6, 7). The CAR transgene is inserted into T cells, enabling them to recognize antigens, deplete malignant cells in a non-HLA-dependent manner, and function as living drugs.

Recent research has explored the development of CAR T cells to identify and eliminate senescent cells just in preclinical models. Yet, their application in the elderly human population presents significant challenges (8). This review aimed to summarize the relationship between senescence and CAR T cell therapy, including how senescence may affect the efficacy of CAR T therapy and the potential use of CAR T cells for the treatment of age-related diseases.

## 2 CAR T cell design

Since the 1990s, five generations of CAR T cell therapies have been developed. The first generation consisted solely of single-chain variable fragment (scFv) binding to the CD3ζ chain for T cell activation but lacked clinical efficacy, since exhibited a limited proliferation and cytotoxicity. The second and third generation integrates costimulatory domains to enhance the expansion and long-term survival of genetically engineered cells *in vivo*, second generation includes only one, CD28 or 4-1BB, whereas the third generation includes both (tuning) (9).

Carle June pioneered the administration of CAR T cells to patients with chronic lymphocytic leukemia (10) and acute lymphoblastic leukemia (ALL) (11). In 2017, Tisagenlecleucel and Axicabtagene ciloleucel became the first CAR T cell therapies approved by the FDA, demonstrating promising results in phase II studies. Tisagenlecleucel showed an overall response rate of 81% in pediatric and young adult patients with ALL (12), whereas axicabtagene ciloleucel achieved a complete remission rate of 82% in patients with refractory large B-cell lymphoma (13).

**Figure 1:**
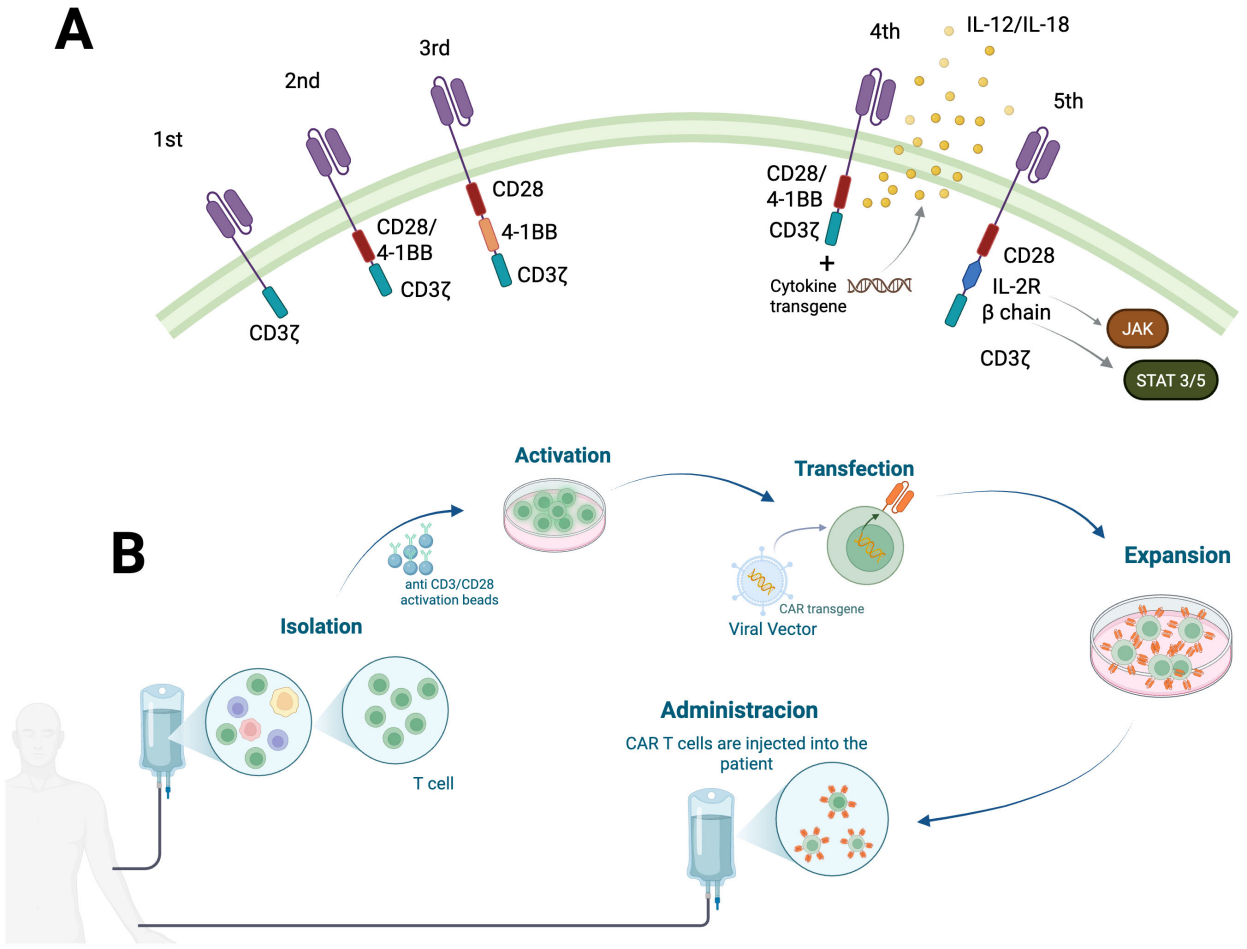
CAR T cell building. **(A)** The structural composition of CAR demonstrates a consistent architecture comprising four domains across all five CAR generations. **(B)** General procedures for CAR T cell manufacturing.

Nevertheless, the effectiveness of CAR T cells in treating solid tumors remains a significant challenge due to several factors, including the limited ability of CAR T cells to infiltrate tumors, tumor antigen heterogeneity, and the inhibitory tumor microenvironment (14). To address these challenges, enhancer designs in fourth-generation CAR T cells co-express vectors encoding cytokines or others immunomodulatory proteins, which enable T cells to stimulate cytokine release within the tumor microenvironment, and sustain T cells responses. This new category of cells is also referred to as T cell redirected for antigen-unrestricted cytokine-initiated killing (TRUCKs). TRUK-T cells often produce one or more type of cytokines, including IL-12, IL-18, IL-15, IL-2, IL-23, and IL-21, and have been tested in solid tumors such as pancreatic, lung, and colorectal tumors, showing the capacity to enhance the infiltration and cytotoxicity of CAR T cells (15). The fifth generation features an IL-2R β chain and a STAT3-binding tyrosine-X-X-glutamine (YXXQ) motif, which simulates the JAK kinase, STAT3 and STAT5 pathways, thereby improving CAR T cell longevity, proliferation, and persistence in both liquid and solid tumors models (**Figure 1A**) (16).

Furthermore, inhibitory or modulatory elements have been developed to mitigate the suppressive tumor microenvironment. The administration of antibodies that inhibit the Programmed cell death protein 1 (PD-1), a recognized inhibitory pathway expressed on tumor cells, has demonstrated enhanced efficacy of CAR T-cell therapy in both preclinical and clinical contexts (17). Another inhibitory approach involves genetic engineering strategies, such as the generation of PD-1 and TCR deficient mesothelin-specific CAR-T (MPTK-CAR-T) cells, the deletion of CTLA4, and the inactivation of TGF-β signaling through a dominant-negative TGF-β receptor II (14).

The manufacturing and application of different CAR T cells generally involves the following steps: 1) T cell isolation. 2) T cell activation using beads coated with antibodies or plate-bound antibodies, with anti-CD3/anti-CD28 antibodies being the most commonly employed. 3) Genetic modification to insert recombinant DNA coding for the CAR gene and possibly additional genes using lentivirus, gamma-retrovirus, or other methods. 4) The expansion of genetically modified T cells in culture to exponentially increase the number of cells for clinical intervention. 5) Cryopreservation, lymphodepletion, and CAR T cell infusion (**Figure 1B**) (18).

## 3 Senescence and its clinical implications

Cellular senescence is a process in which cell proliferation is inhibited due to different factors including telomere shortening, DNA damage, oxidative stress, oncogenic activation, loss of tumor suppressor genes, mitochondrial dysfunction, and ionizing radiation or drugs, leading to irreversible cell cycle arrest (19). Once the cells are senescent, they secrete biologically active agents referred to as SASP, which affect cell function. The SASP primarily consists of chemokines and cytokines, including IL-6, IL-8, and members of the CC-chemokine ligand family such as CCL2 and CCL5, as well as growth factors such as growth-regulated oncogene (GRO), hepatocyte growth factor (HGF), and insulin-like growth factor–binding proteins (IGFBPs), matrix metalloproteinases(MMPs) that break down the extracellular matrix, Reactive Oxygen and Nitrogen Species (ROS/RNS) (20) (**Table 1**). Importantly, SASP-associated ROS not only contribute to chronic inflammation and macromolecular damage but can also reinforce senescence by inducing DNA damage, thereby facilitating the propagation of senescence to neighboring cells (21).

**Table 1:** Main components of the senescence-associated secretory phenotype (SASP).

Category	Example SASP mediators	Main pathological impacts
Cytokines/Interleukins	IL-6, IL-8 (CXCL8), IL-1α, IL-1β, IL-11, TNF-α, GM-CSF	Chronic inflammation, paracrine senescence, immune cell recruitment/activation, systemic inflammation
Chemokines	CXCL1 (GROα), CXCL2, CXCL3, CXCL10, CXCL14, CCL2, CCL5	Recruit monocytes/macrophages and neutrophils; promote tumorigenesis, tissue remodeling, immune infiltration
Growth factors	TGF-β, HGF (hepatocyte growth factor), IGFBP-7, Amphiregulin (AREG), VEGF	Fibrosis and ECM deposition (TGF-β), proliferation and EMT, metabolic dysregulation, angiogenesis
Proteases/ECM modulators	MMP1, MMP3, ADAM family proteases	Extracellular matrix degradation, tissue remodeling.
Other mediators/signals	ROS/RNS, extracellular vesicles (EVs), IGFBPs	Oxidative damage, paracrine transfer of senescence signals, metabolic effects

The SASP contributes mechanistically to chronic disease through various pathways. For instance, SASP cytokines and MMPs promote endothelial dysfunction, enhance adhesion molecule expression, degrade the extracellular matrix, and facilitate leukocyte infiltration. These events accelerate the progression of atherosclerosis, vascular stiffening, and microvascular rarefaction (22). Furthermore, in fibrotic lung disease and other fibrotic disorders, the continuous secretion of MMPs and matrix-modifying factors by senescent cells results in aberrant extracellular matrix turnover, excessive collagen deposition, loss of elasticity, and fibrosis (23).

Replicative senescence is one of the most extensively studied forms, primarily attributed to a DNA damage response induced by telomere shortening in proliferating cells. Normal human diploid cells have a finite capacity for division, reaching their Hayflick limit. Telomeres lose approximately 50–250 bp per mitosis, leading to erosion. While telomerase mitigates shortening, though, most of somatic lack adequate telomerase to maintain telomere length, leading shortening with age (19).

Conversely, the SASP also plays beneficial roles, notably in tissue repair and remodeling, by recruiting immune cells to facilitate these processes (24). For example, the reduction of senescent cells in neonatal mice, achieved through either pharmacological or genetic interventions, has been observed to disrupt normal lung development (25). Cellular senescence can be induced by damage or stress, subsequently utilizing the SASP to facilitate healing and tissue remodeling processes (24).

Cellular senescence is a double-edged sword in cancer research. In principle, senescence is a natural barrier to tumorigenesis, arresting the proliferation of damaged or potentially malignant cells. In parallel, SASP-mediated recruitment of immune and stromal cells supports tissue repair and remodeling, which also contributes to the transient protective effect against early tumor development. Senescence is therefore considered, at least in part, a cancer suppression response, as carcinogenic mutations can induce oncogene-induced senescence (OIS) in cells via at least two tumor suppressor pathways: p21/p53 and the pRb/p16Ink4a signaling pathways. In contrast, the induction of senescence in cancer cells by ROS is primarily dependent on p16, p21, and/or p27. The main mechanism behind OIS is the DNA damage response. The increase in irreparable DNA damage leads to the activation of checkpoint kinase 2 (CHK2) and ataxia telangiectasia mutated (ATM), resulting in the phosphorylation of histone H2AX and p53, and subsequently activating the p53–p21 pathway. These mechanisms inhibit the transmission of mutations to subsequent cellular generations and enhance the efficiency of immune system clearance, thereby mitigating the risk of tumor development (21, 26). The OIS initiates a robust immune, mediated clearance mechanism through the recruitment of natural killer cells, macrophages, and cytotoxic T lymphocytes, driven by the SASP. However, this immunosurveillance becomes increasingly compromised with advancing age. The age-related decline in effector functions, such as diminished NK cell cytotoxicity and T-cell proliferative capacity, along with the dysfunction of myeloid phagocytes and the emergence of senescent or suppressive immune subsets, impairs the recognition and elimination of senescent targets, thereby facilitating their persistent accumulation (27). Furthermore, senescent cells actively evade clearance by upregulating anti-phagocytic and anti-apoptotic mechanisms, such as the CD47-QPCT/L axis and senescent cell anti-apoptotic pathways. This exacerbates the age-associated failure of immunosurveillance and links OIS to chronic tissue dysfunction (28).

Senescent cells play key roles in physiological processes, such as tumor suppression, wound healing, and tissue fibrosis; however, their accumulation in tissues due to illnesses or aging promotes tissue dysfunction and the aging phenotype, leading to chronic diseases and age-related illnesses.

## 4 Immunosenescence: how senescence affects T lymphocytes

The aging process compromises multiple cellular systems through mechanisms such as genomic instability and epigenetic alterations that drive loss of proteostasis, down regulation of metabolism, cellular senescence, mitochondrial dysfunction, stem cell depletion, and compromised intercellular communication (29). Among the primary age-related changes affecting immunity are thymic involution and reduced cellularity and proliferative capacity within the bone marrow (30). The thymus, the central organ responsible for generating self-tolerant T cells, undergoes early and progressive atrophy, becoming largely nonfunctional by mid-adulthood. As thymopoiesis declines, the production of naïve T cells falls sharply, leading to a peripheral T-cell compartment increasingly dominated by memory and exhausted phenotypes (31). Histological analyses further reveal a disrupted thymic architecture characterized by reduced thymic epithelial cells, loss of clear cortico-medullary demarcation, and progressive replacement of functional tissue with adipocytes and fibroblasts (32). In parallel, aging skews hematopoietic stem cells toward a myeloid-biased differentiation program, reducing lymphoid progenitors and ultimately diminishing B-cell precursor output (33). Collectively, these alterations impair innate and adaptive immunity, resulting in a broader decline known as immunosenescence (31, 34).

Immunosenescence is characterized by alterations in the naïve-to-memory T-cell ratio, decreased CD4:CD8 ratio, impaired calcium-mediated signaling, and thymic atrophy (35, 36). Functionally, Treg cells expand and increase suppressive activity, while CD8^+^ T cells lose naïve subsets and accumulate CD28^+^ effector-memory populations with poor proliferative capacity, altered cytokine secretion, and heightened expression of senescence-associated markers such as CD57 and KLRG-1. B-cell generation and antibody quality also decline, with an enrichment of age-associated B cells expressing T-bet that promote autoreactivity and weaken humoral defenses (31). Many immune cell types additionally exhibit age-related decline and increased expression of p16^INK4a^ (also known as CDKN2A) and p21^CIP1^ (CDKN1A), reinforcing cell cycle arrest triggered by replicative stress or DNA damage (35, 36).

Undoubtedly, a bidirectional axis of deterioration is established between chronic degenerative processes and immune system effects, which could explain how aging plays an important role in the generation of hallmark aging and senescent lymphocytes (30). Recent findings involving genetic mutations in T cells, such as the T lymphocyte-specific knockout of mitochondrial transcription factor A (TFAM), reveal that these alterations not only impair metabolism and drive T cells to senescence, but also result in cognitive, metabolic, and physical harm. Moreover, they can cause cardiovascular alterations that ultimately lead to premature death (37). Concurrently, aging sustains a low-grade proinflammatory state—known as inflammaging—driven by the accumulation of senescent cells with a pro-inflammatory secretory profile, metabolic disturbances, and microbiome alterations (31). Immunosenescence and inflammaging function as interconnected pillars of immune aging: the former diminishes immune surveillance and functionality, while the latter perpetuates chronic immune activation. Together, they form a self-reinforcing loop that accelerates systemic dysfunction and amplifies age-related pathology (31). In particular, KLRG-1^+^ or CD57^+^ expression in T cells is associated with decreased effector cytokine production, poor proliferation, and increased susceptibility to apoptosis (38, 39).

As exhaustion and senescence are distinct states of T-cell dysfunction, they exhibit several overlapping characteristics, including impaired proliferation and cell cycle arrest. Nonetheless, intrinsic characteristics, including the expression of specific surface receptors and distinct cytokine secretion profiles, can be used to distinguish between these states.

Although exhaustion and senescence represent distinct forms of T-cell dysfunction, they share features such as impaired proliferation and cell cycle arrest. They can, however, be differentiated by surface receptor profiles and cytokine secretion patterns (40). T-cell exhaustion arises from chronic antigenic stimulation and reflects a regulatory state that limits effector activity. This phenotype is shaped by defined transcriptional and epigenetic programs, including increased expression of inhibitory receptors such as PD-1 and LAG-3 and induction of transcription factors like TOX and the NR4A family, which collectively enforce a hyporesponsive profile (41). Replicative senescence, in contrast, results from cumulative telomere shortening and ultimately leads to irreversible growth arrest accompanied by the loss of costimulatory receptors, such as CD27 and CD28, and an increase in co-inhibitory receptors, including KLRG-1, CD57, and TIM-3 (42). Aging also drives a marked contraction of the TCR repertoire, characterized by oligoclonal expansions and reduced diversity stemming from diminished thymic output and homeostatic proliferation, ultimately limiting the antigenic breadth and functional adaptability of T cells available for CAR-T manufacturing (43). At the epigenetic level, age-associated remodeling captured by DNA-methylation aging clocks—such as acceleration of DNAmAge signatures—further reflects cumulative transcriptional drift and fixed chromatin states that impair T-cell plasticity, responsiveness, and proliferative potential (44).

These cumulative alterations in T-cell composition, proliferative capacity, metabolic fitness, and transcriptional programming directly limit the quality of lymphocytes available for therapeutic engineering, thereby imposing critical constraints on both the generation and functional performance of CAR-T cell products in aging individuals (45). At the metabolic level, aged T cells display compromised mitochondrial function, reduced spare respiratory capacity, and a shift toward dysfunctional glycolytic engagement, all of which impair their ability to sustain the bioenergetic demands required for robust CAR-T expansion and long-term persistence (46). Recent clinical observations support this age-dependent decline in product fitness: older lymphoma patients consistently exhibit reduced CAR-T peak expansion and attenuated persistence, associated with enriched frequencies of senescent CD27^-^CD28^-^ and PD-1^+^LAG-3^+^ T-cell subsets in the leukapheresis material (47, 48). Likewise, CAR-T products manufactured from aged donors demonstrate impaired proliferative indices, mitochondrial dysfunction, and increased exhaustion signatures, which correlate with diminished *in vivo* expansion and inferior clinical responses. In this context, higher frequencies of senescent or exhausted T cells—marked by loss of CD27/CD28 (48) or increased expression of PD-1, LAG-3, TIGIT, or TOX—have been consistently associated with reduced CAR-T expansion, poorer persistence, and inferior clinical responses, underscoring immunosenescence as a key determinant of CAR-T therapy outcomes (49).

## 5 CAR T cells therapy and age-related pathologies

Recently, immunotherapy has emerged as a novel senolytic strategy for the treatment of senescence-associated pathologies. Amor et al. pioneered the use of CAR T cells to eliminate senescent cells. Initially, they sought a receptor specific to senescent cells in three senescence models: therapy-induced senescence in murine lung adenocarcinoma, oncogene-induced senescence in murine hepatocytes, and culture-induced senescence in murine hepatic stellate cells (HSCs). They identified the urokinase plasminogen activator receptor (uPAR), which facilitates degradation of the extracellular matrix. In human liver fibrosis tissues, an increase in uPAR was confirmed, co-expressing with p16 and IL6, which are markers of senescence. CAR T cells targeting uPAR were developed, comprising anti-murine uPAR (scFv) linked to the CD28 costimulatory and CD3ζ signaling domains. uPAR-targeted CAR T cells have demonstrated efficacy in eliminating senescent cells both *in vitro* and *in vivo*. Specifically, CAR T cells prolong the lifespan of mice bearing lung adenocarcinoma and depleted HSCs in murine models of liver fibrosis induced by CCL_4_ and diet (50).

Notably, these uPAR-directed CAR T cells have been tested in young animals with experimentally induced senescence-related pathologies (50). In 2024, Amor et al. identified an accumulation of uPAR-positive senescent cells in aged human tissues, particularly in pancreatic tissues, from individuals aged 50–76 years. In naturally aged mice (20 months), an increase in uPAR within senescent cells was observed. The infusion of anti-uPAR CAR T cells to target these cells resulted in a decrease in the number of cells positive for beta-galactosidase (SA-β-gal), the most used biomarker for senescent cells, along with a reduction in proinflammatory cytokines. Additionally, the authors demonstrated improvements in metabolic parameters in mice fed a high-fat diet and treated with anti-uPAR CAR T cells (51). More recently, uPAR-specific CAR T cells have shown the ability to eliminate senescent cells in aging and injured gastrointestinal tract. In 20-month-old murine models, anti-uPAR CAR T cells reduced intestinal permeability and increased epithelial cell proliferation in the crypts. Similarly, in a colitis mouse model, these CAR T cells mitigated weight loss and reduced the severity of intestinal injury. Remarkably, both *in vivo* and *in vitro* studies indicated that anti-uPAR CAR T cells can enhance the regenerative capacity of aged intestinal stem cells (52). Notably, none of these three studies reported toxicity induced by CAR T cell therapy, such as cytokine release syndrome.

A different explored receptor for targeting senescent cells is the natural killer group 2 member D ligands (NKG2DLs). anti-NKG2D CAR T cells were first developed to target glioblastoma and cancer stem cells (53). The receptor comprises the extracellular domains of mouse NKG2D, mouse CD137, and mouse CD3ζ signaling domains. Furthermore, since NKG2DLs are highly expressed in senescent cells but not in normal cells, Yang et al. showed the efficacy of NKG2D CAR T cells in removing senescent cells induced by DNA damage, oncogenic and replicative stress, or overexpression of P16^INK4a^ (*in vitro* models). Remarkably, senescent cells were also deleted in both irradiated and aged mice as well as in aged non-human primate models without any reported adverse effects (54). The senolytic efficacy of NKG2D-CAR T cells was further corroborated by Deng et al. in stress-associated senescent cell lines. The authors utilized brain cell lines, specifically forebrain astrocytes and embryonic fibroblasts, in which senescence has been associated with cognitive decline and diseases such as Alzheimer’s disease (55).

Although uPAR- and NKG2D-targeted CAR-T cells yield robust senolytic effects in preclinical models, several translational barriers must be addressed before human application.

Despite significant progress in characterizing the senescent phenotype using advanced molecular biology techniques, senescent cell heterogeneity represents a major challenge in identifying a universal antigen shared among them. This obstacle poses a significant hurdle in the application of CAR T cells for the elimination of senescent cells. Consequently, it is imperative to investigate alternative potential targets for developing effective senolytic CAR T cells. Using transcriptomic and proteomic data, Dent et al. identified 38 surface proteins that were highly expressed in senescent cells across 13 different tissues, which could potentially serve as viable targets for CAR T cells (56).

Another crucial aspect, as previously mentioned, is that senescence also plays beneficial roles, and its elimination can have detrimental consequences. In a recent study, pharmacological elimination of senescent HSCs using the senolytic agent ABT26 or through genetic ablation, significantly impaired liver regeneration after partial hepatectomy in young mice. The authors noted that the SASP induces liver regeneration, specifically through the secretion of IL-6 and CXCR2 ligands (57), highlighting that not all senescent cells are pathological and that their complete removal might disrupt vital repair processes.

An additional critical challenge involves the tissue penetration and trafficking of CAR T cells. While preclinical studies provide clear evidence of CAR-T cell accumulation in target tissues, such as the uPAR-directed constructs described by Amor et al., where anti-uPAR CAR T cells accumulated around senescent hepatocytes within seven days post-infusion, and exhibited an effector-memory phenotype with low exhaustion markers, thereby supporting effective tissue infiltration in these murine models (50), several biological and biophysical characteristics of aged or fibrotic human tissues suggest that achieving comparable infiltration and distribution of CAR-T cells may be more challenging in patients than in experimental mouse models. Particularly, fibrotic and aged tissues accumulate cross-linked collagen, glycosaminoglycans and advanced glycation end-products, which contribute to increased matrix stiffness and reduced interstitial porosity. These changes create steric barriers that may impede the movement of T cells through the parenchyma (58). Immunomodulatory factors such as TGF-β and CXCL12 may also be present, contributing to a chemokine environment that excludes effector T cells. TGF-β signaling has the potential to downregulate T-cell chemokine receptors and inhibit cytotoxic functions (59).

On the other hand, the application of CAR T cell therapy has demonstrated clinical benefits, however approximately one-third of patients may experience systemic inflammatory toxicity, known as cytokine release syndrome (CRS), due to robust immune activation. Upon antigen recognition, CAR T cells secrete pro-inflammatory cytokines, such as IFN-γ and GM-CSF, which activate myeloid cells to further amplify the inflammatory cascade by producing additional cytokines, including IL-6 and IL-1 (60). While none of the preclinical senolytic CAR-T studies reviewed herein reported overt toxicity (**Table 2**), this does not eliminate the possibility of adverse effects arising in human patients. Indeed, an increasing body of evidence underscores that conventional murine xenograft models inadequately predict human-specific toxicities, as many human tissue-specific antigens are not conserved in mice (61).

**Table 2:** Summary of preclinical CAR T cell strategies targeting senescent cells.

Target	Key study (ref)	Models used	Main outcomes	Safety/toxicity findings
anti-uPAR	Amor C et al., *Nature* 2020. (50)	*In vitro* senescent cell killing; murine models: therapy-induced senescence in lung adenocarcinoma, oncogene-induced hepatocyte senescence, culture-induced senescent hepatic stellate cells; liver fibrosis models (CCl_4_ + diet).	uPAR-CAR T cells selectively eliminated senescent cells, depleted senescent HSCs in fibrotic livers, prolonged survival in lung adenocarcinoma model; improvement of fibrosis markers and tissue function.	No overt CAR-T-related toxicity reported in murine models (no CRS reported) in this study.
	Amor C et al., *Nat Aging* 2024. (51)	Aged human tissue analysis (uPAR+ senescent cells); naturally aged mice (20-month), high-fat diet (HFD) mice.	Single infusion of anti-uPAR CAR T reduced SA-β-gal+ cells, pro-inflammatory cytokines, improved metabolic parameters (glucose tolerance), exercise capacity.	No CAR-T associated CRS or severe toxicity reported in the mouse models.
	Eskiocak O et al., bioRxiv 2024. (53)	*In vitro* assays; 20-month-old murine, colitis models; intestinal stem cell assays.	Anti-uPAR CAR T reduced intestinal permeability, increased crypt epithelial proliferation, improved ISC regenerative capacity, mitigated weight loss and injury severity in colitis.	No CRS or systemic toxicity reported in treated mice in this study.
anti-NKG2D	Yang D et al., *J Immunother Cancer* 2019. (53)	*In vitro* glioblastoma cells/cancer stem cells; murine xenografts of glioblastoma.	NKG2D-CAR T efficiently lysed glioblastoma cells and stem cells *in vitro*, eliminated xenograft tumors *in vivo*.	No significant treatment-related toxicity reported in the treated mice; proliferation, apoptosis, and genomic stability of CAR T cells unaffected.
	Yang D et al., *Sci Transl Med* 2023. (54)	*In vitro* senescent cell lines; aged mice; aged non-human primates (NHPs).	NKG2D-CAR T removed senescent cells induced by DNA damage/oncogenic/replicative stress, in aged mice and NHPs.	No adverse effects observed in aged NHPs in this report; clinical safety not yet established.

Finally, fundamental challenge for senolytic CAR-T therapy is that ageing not only leads to accumulation of senescent target cells but also to functional decline of the immune system — a phenomenon known as immunosenescence.

## 6 Effect of senescence on CAR T cell efficacy

Given that the therapeutic activity of CAR T cells ultimately depends on their ability to reach the target tissue and maintain direct, sustained interactions with pathogenic cells, it is essential to recognize that their functional decline results from a combination of intrinsic and extrinsic influences. Intrinsic factors—including activation-driven exhaustion, accelerated differentiation, and the gradual emergence of senescent traits—are largely unavoidable once CAR T cells repeatedly engage antigen *in vivo*. At the same time, extrinsic pressures within the surrounding tissue microenvironment—such as nutrient competition, suppressive cytokines, stromal constraints, and chronic inflammatory signals—further compromise their persistence and cytotoxic performance. Together, these forces push the population toward a state marked by reduced proliferative capacity and diminished effector activity, in contrast to the superior clinical outcomes associated with preserving a stem-cell–memory-like phenotype. For this reason, a precise characterization of the differentiation status and baseline fitness of T cells before their genetic engineering becomes a critical step in optimizing CAR T-cell therapies across diverse pathological contexts (42, 62).

### 6.1 Intrinsic factors limiting CAR T-cell fitness and potential approaches to mitigate them

The intrinsic programs that arise within CAR T cells after repeated antigen stimulation constitute a major barrier to sustained therapeutic activity. As these cells proliferate and engage their targets, they progressively acquire dysfunctional features—including exhaustion, terminal differentiation, and senescent traits—that compromise proliferative capacity, cytokine secretion, and long-term persistence. Although these alterations originate from the cellular stress imposed on the CAR T compartment rather than from the external milieu, several strategies have begun to address the molecular pathways that drive this decline (63).

Senescence is no longer regarded as an irreversible state, highlighting the potential of molecular tools designed to block the critical signaling pathways involved in its development. Such advancements hold significant promise for enhancing the efficacy of CAR T cell therapy. Recent studies have demonstrated that activation of the CD28 signaling pathway, combined with co-stimulation via mutant CD28 constructs in CAR T cells, can restore their immunocompetence. This approach has been shown to improve long-term persistence within the tumor microenvironment, thereby enhancing therapeutic potential (64, 65).

Another strategy under investigation is the simultaneous blockade of the immune checkpoints PD-1, TIM-3, and LAG-3. This combinatorial approach demonstrated a synergistic effect potentiating the effector functions of CAR T cells (66). In addition to the antibody-based blocking of Galectin-9, a putative TIM-3 ligand, this approach has been shown to reduce CAR T cell exhaustion and significantly enhance cytotoxicity against previously resistant tumor cells (67). Together, these observations illustrate how selectively targeting intrinsic senescence- and exhaustion-related pathways may bolster the functional durability of CAR T-cell products without altering their fundamental design.

### 6.2 Extrinsic factors shaping CAR T-cell exhaustion and senescence and approaches under exploration

Extrinsic pressures arising from the surrounding tissue microenvironment further accelerate CAR T-cell dysfunction. Hypoxia, nutrient competition, suppressive cytokines such as TGF-β and IL-10, and interactions with regulatory cell populations contribute to the deepening of exhaustion and senescence programs. Although these factors lie outside the intrinsic biology of the infused cells, several interventions have been explored to counteract them, including metabolic reprogramming strategies—such as adenosine-targeting through ADA overexpression—and approaches designed to limit inhibitory ligand engagement. While these tools remain adjunctive, they highlight how modulation of the microenvironment may complement efforts to preserve CAR T-cell functionality (68).

Adenosine is a metabolite released during inflammation and hypoxia that downregulates CD28 expression, induces apoptosis, and accelerates replicative senescence by reducing telomerase activity. Its blockage can lead to increased T cell fitness (69). Additionally, modifying CAR T cells overexpressing ADA augmented CAR T cell exhaustion resistance, another promising strategy (70).

Molecular tools to reverse senescence undoubtedly represent one less obstacle and an advancement in CAR T cell engineering, yielding promising results and improving the safety of their manufacturing and treatment. However, risks remain, including unexpected toxicity or cytokine storms resulting from overactivation or prolonged activity owing to these modifications. This highlights the necessity for further research and the development of modifications to ensure the intended therapeutic outcomes without compromising patient safety.

## 7 Future perspectives

Most senolytic agents suppress anti-apoptotic pathways, potentially leading to unintended consequences in normal tissues. CAR T cells provide a more precise approach for targeting specific antigens on the cell surface.

As previously described, CAR T cells have been explored in certain senescence-associated diseases; however, their potential applications extend to a broader spectrum of conditions. For instance, senescent T cells have been used as therapeutic targets for obesity treatment. Senescent T cells were characterized as CD4^+^ CD44^high^ CD62^low^ PD-1^+^ CD153^+^. Yoshida et al. utilized CD153 vaccination, another immunotherapy approach, to eliminate senescent T cells from visceral adipose tissue. In mice fed a high-fat diet to induce obesity, the authors observed a decrease in adipose-senescent T cells and an improvement in obesity-related features (71). It is important to highlight that the elimination of senescent T cells aims to restore immune competence and may indirectly enhance CAR T cell fitness, expansion, and persistence, particularly in older individuals. In a similar study, a vaccine targeting glycoprotein non-metastatic melanoma protein B (GPNMB), which is highly expressed in senescent vascular endothelial cells in mice with atherosclerosis, attenuated senescence, reduced atherosclerotic burden, and increased lifespan (72). In addition, Aguayo-Mazzucato et al. demonstrated that senescent pancreatic β-cells were induced by insulin resistance, and their removal using a senolytic agent (ABT263) or transgenic INK-ATTAC mice increased insulin secretion and glucose homeostasis (73). These studies indicate that the elimination of senescent cells represents a potential therapeutic approach for various diseases, wherein CAR T cells may be utilized.

Furthermore, uPAR-specific CAR T cell therapy could be a potential treatment for other hepatic senescence-related pathologies, such as metabolic dysfunction-associated steatohepatitis (MASH). HSCs are activated in response to liver injury and transform from a quiescent state into myofibroblasts, which are the major producers of extracellular matrix that accumulate as fibrosis and contribute to hepatocellular carcinoma (HCC) pathogenesis (74).

Recently, Yashaswini et al. showed that uPAR is expressed in activated senescent HSCs in human and murine models of MASH. However, it is also expressed in activated HSCs, immunosuppressive Trem2^+^ macrophages, and polymorphonuclear myeloid-derived suppressor cells (PMN-MDSCs). Therefore, the authors sought other more specific markers, proposing CD206, encoded by MRC1, which is more restrictive for senescent HSCs in human and murine MASH livers (75), opening the door to the development of CAR T cells for the treatment of MASH and fibrosis-related diseases.

CAR T cells have already been engineered to target activated cardiac fibroblasts, which deposit excess extracellular matrix in the myocardium and demonstrate a significant reduction in cardiac fibrosis in mice (76). Recently, a novel strategy for cardiac fibrosis was developed to overcome the key limitations of CAR T cells in non-oncological diseases, ensuring that cell depletion occurs exclusively in the target tissue. Rurik et al. generated transient CAR T cells *in vivo* using lipid nanoparticles (LNPs) targeting CD5 to deliver mRNAs to T Cells. CAR mRNA was developed to target fibroblast activation protein (FAP), a biomarker of activated fibroblasts used to treat cardiac injury. This approach yielded efficacious CAR T cells *in vivo* and mitigated fibrosis while restoring cardiac function. This mRNA-LNP–mediated *in vivo* generation of CAR T cells signifies a transition from the intricate ex vivo manufacturing process to a transient, “off-the-shelf” immunotherapy, which can be administered and repeated similarly to mRNA vaccines. This advancement extends CAR T technology beyond applications in senescence or fibrosis, facilitating immunomodulation in diseases that necessitate transient, tissue-specific intervention (77).

Establishing robust and context-specific senescence phenotypes is crucial for the safe and effective implementation of CAR T-cell therapies targeting senescent cells. Recently, the SenNet Consortium has launched a large-scale initiative to generate a multimodal atlas of senescent cells across human and mouse tissues throughout the lifespan, integrating single-cell transcriptomics, spatial proteomics, chromatin organization profiling, secretome analyses, and 3D genomics. Through these coordinated efforts, the consortium aims to define tissue-specific and context-dependent senescent cell subtypes (78). Achieving this level of resolution is vital for designing next-generation CAR T cells capable of distinguishing pathological senescent cells from those that play beneficial roles in tissue repair or remodeling. Consequently, advancing and integrating multiomic senescence profiling should be prioritized in the future development of senescence-directed cellular immunotherapies.

Additional significant challenges for applying CAR T cells to age-related pathologies include the absence of lymphodepletion, which is commonly used in oncological CAR T cell treatments prior to infusion. Another important factor is that CAR T cell therapy has primarily been administered to younger individuals, while senescence-related diseases mostly affect the elderly (8). This contrast raises concerns about impaired T cell fitness and cellular senescence in older patients, highlighting the importance of evaluating immunological status and T cell phenotype before infusion. Biomarkers such as CD27, CD28, TIM-3, CCR7, PD-1, and LAG-3 could help identify dysfunctional or senescent T cells (42, 79). Additionally, DNA methylation signatures serve as potential biomarkers for determining biological age in specific tissues and cells (80). The integration of these tools with extensive clinical evaluation may enhance the determination of patient eligibility.

Several strategies have been tested to induce T cell rejuvenation and mitigate the risks associated with the use of CAR T cells in adult individuals. Calorie restriction and physical activity enhanced T cell fitness in patients (81). Additionally, the use of allogeneic transplants, which involve employing young T-cell donors to produce CAR T cells, was explored; however, this approach was associated with the development of graft-versus-host disease (82). Furthermore, pretreatment of T cells with rapamycin attenuated mTORC1 activity and upregulated CXCR4 expression, thereby augmenting their migration within the bone marrow (83).

Although there is promising research on the potential of CAR T cell therapy to treat senescence-associated pathologies, the number of studies is limited, and more evidence is needed to translate the results from animal models to clinical human trials.

## 8 Conclusion

The application of CAR T cells in non-oncological diseases, specifically senescence-related diseases, is a growing area of research. Although there are still significant limitations, such as identifying specific receptors for senescent cells in target tissues and the high cost of their application, many senescence-related diseases currently lack effective treatments. In this context, CAR T cells present distinct advantages over pharmacological senolytics, notably their high specificity, adaptability, and the potential for reprogramming to recognize complex senescent phenotypes.

**Figure 2:**
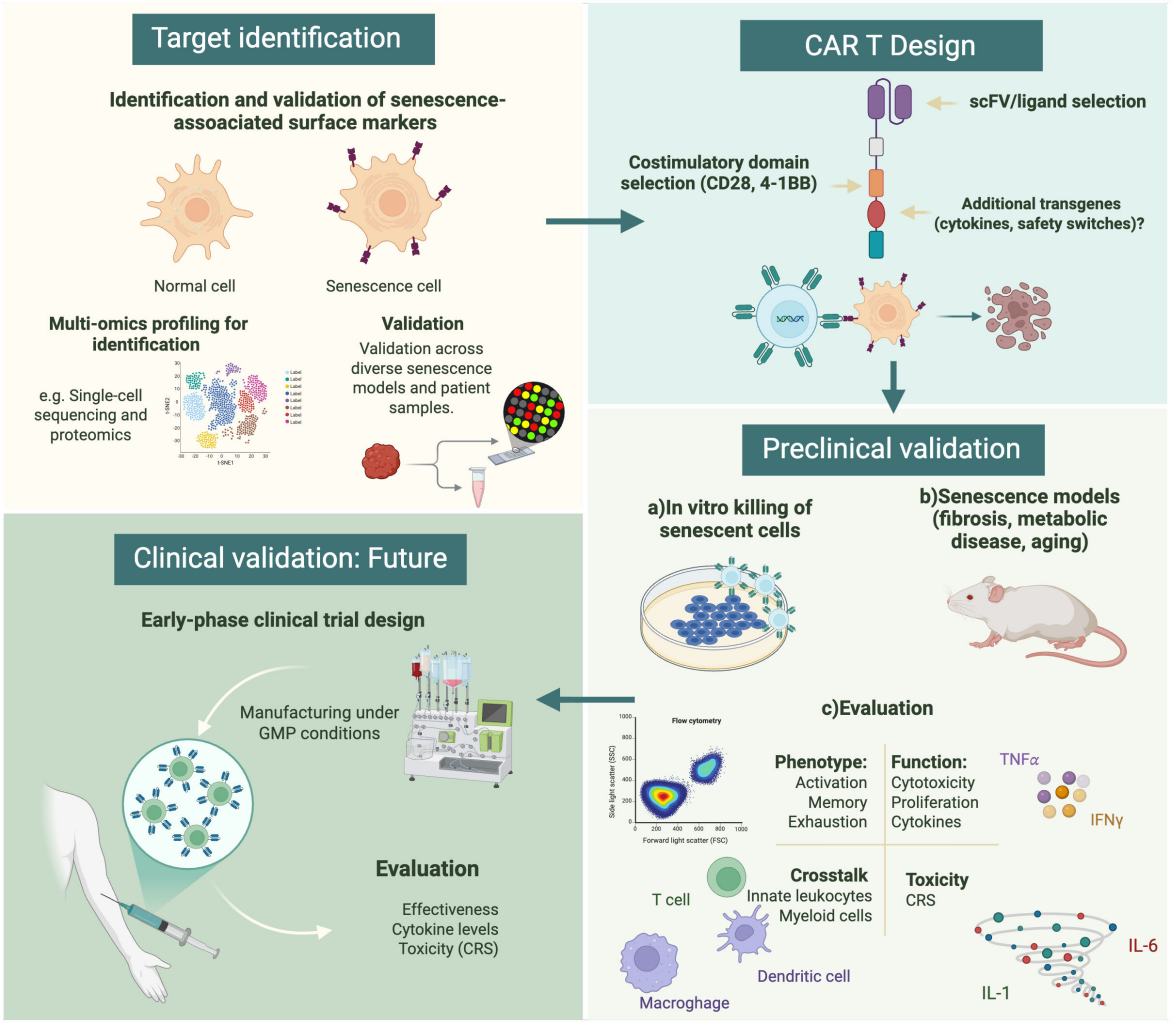
Conceptual workflow for the translational development of senescence-targeted CAR T cell therapies. Initially, it is crucial to identify senescence-associated surface markers using multi-omics approaches, followed by their validation across diverse senescence models and patient samples. Subsequently, during the CAR T design stage, single-chain variable fragments (scFvs) and costimulatory domains (e.g., CD28, 4-1BB) will be selected. Additionally, the incorporation of transgenes such as cytokines or safety switches could represent a significant improvement in future designs to enhance their effectiveness and reduce off-target effects. Preclinical validation encompasses *in vitro* cytotoxicity assays, evaluation in senescence-associated disease models (e.g., fibrosis, metabolic disease, aging), and comprehensive immunophenotyping and potential toxicity assessment. Finally, although not yet realized, future early-phase clinical development will require GMP manufacturing and the assessment of safety, cytokine kinetics, and preliminary therapeutic activity in human subjects.

To realize the application of CAR T-cell therapy for age-related pathologies, several future research priorities must be addressed. These priorities include the validation of cell surface markers capable of reliably identifying senescent cells, the investigation of strategies to rejuvenate or modulate senescent T cells to enhance therapeutic efficacy, and the development of scalable and cost-effective manufacturing platforms (**Figure 2**). Addressing these challenges will facilitate the translation of senolytic CAR T cell therapy into safe, practical, and widely accessible interventions for age-related and chronic diseases.
